# Psychological Consequences of Breast Cancer in Iran: A Meta-Analysis

**Published:** 2019-05

**Authors:** Nasrin REFAEE SAEEDI, Hamidreza AGHAMOHAMMADIAN SHARBAF, Mohammad Javad ASGHARI EBRAHIMABAD, Hossein KARESHKI

**Affiliations:** 1. Department of Psychology, Faculty of Education and Psychology, Ferdowsi University of Mashhad, Mashhad, Iran; 2. Department of Clinical Psychology, Faculty of Education and Psychology, Ferdowsi University of Mashhad, Mashhad, Iran

**Keywords:** Psychological consequences, Breast cancer, Iran, Meta-analysis

## Abstract

**Background:**

Breast cancer is known as one of the most common cancers among women and has severe psychological effects. This study aimed to identify the psychological consequences of breast cancer in previous studies based on meta-analysis.

**Methods:**

Meta-analytic procedures were conducted by Prisma guidelines. A literature search was conducted by using following electronic databases including scientific information databases (SID), Magiran, Medlib**,** ScienceDirect, PubMed, Google Scholar, Web of Science, Scopus, CINAHL and Medline from 1991 through 2017 regarding the psychological consequences associated with breast cancer in Iran. The content of all articles was evaluated by the Prisma checklist and analyzed meta-analysis in CMA software.

**Results:**

The final synthesis was carried out on 56 quantitative studies. Considering the findings of meta-analysis of the psychological consequences of patients with breast cancer in seven classes, anxiety (ES=−0.76), body image (ES=0.199), coping strategies (ES= 0.214), depression (ES=−0.700), fatigue (ES=0.322), quality of life (ES= 0.428), and sexual function (ES=0.355) were achieved.

**Conclusion:**

Based on the results of the high level of psychological consequences of breast cancer in women with breast cancer, it is necessary to formulate appropriate therapeutic protocols in order to adjust the psychological consequences.

## Introduction

Breast cancer is known as one of the common cancers among women and is referred to as cancer, which originates from breast tissues, often from milk ducts lining cells (ductal) and lobules around the ducts (lobular) (1). Breast cancer makes up 22.9% of all women’s cancers (2), and in 2016, there are approximately 3.5 million women living with a history of breast cancer in the United States (3). According to the latest statistics from Iran’s Cancer Research Center, about 8500 new cases of breast cancer have been reported annually, and 1400 are death due to breast cancer. Moreover, there are currently about 40000 people living with the disease in the country (4). Breast cancer is a disease that has severe psychological effects. Diagnosis and treatment of cancer are associated with stress and anxiety. Common responses to cancer diagnosis include depression, anxiety, anger, and guilty feelings (5). The incidence of mental disorders in cancer patients is estimated to be between 30% and 40%. About, 80% of cancer patients suffer from significant concern and anxiety in the early stages of their treatment (6–8). The highest mean was related to depression, interpersonal sensitivity, somatization and psychosis regarding nine dimensions of mental health in women with breast cancer (9). Breast cancer patients had higher mean scores in depression and lower mean scores in life expectancy than healthy subjects (8). People with breast cancer experience negative feelings of depression and frustration and have irrational beliefs leading to a poor compromise with the stressful conditions of the disease. Moreover, the majority of these women are not hopeful for the future (10). On the other hand, patients, along with the experience of symptoms of depression and anxiety, commonly use emotion-focused coping strategies that reduce their quality of life (11).

Diagnosis of cancer causes many sense crises in a person. The side effects of treatment in these patients increase emotional disorders and dramatically reduce the quality of life and lead to extensive psychological consequences. However, no research in the country has summarized the studies carried out in this area that could lead to successful clinical decision making. Therefore, given the importance of examining the psychological consequences of breast cancer and its role in the disease process, this study was conducted aiming at examining the meta-analysis of the literature on the psychological consequences of breast cancer in order to help practitioners in designing and implementing interventional plans to prevent and mitigate the negative effects of these consequences and, consequently, to improve the mental status of these patients by recognizing the psychological consequences of this disease.

Hence, the present study was carried out to answer this question: What are the psychological consequences of breast cancer in previous studies based on meta-analysis?

## Methods

This meta-analysis was conducted by the Preferred Reporting Items for Systematic Reviews and Meta-Analyses (PRISMA) Guidelines (12). This literature search was conducted by using the following electronic databases including Scientific Information Databases (SID), Comprehensive Human Sciences Portal, Irandoc, MEDLIB, Noormags, Magiran, Iranmedex database, ScienceDirect, Pubmed, Google Scholar, CINAHL and Medline from 1991 through 2017 regarding the psychological consequences associated with breast cancer in Iran.

### Search Strategy

Searching for articles was accomplished using keywords of mood disorder, anxiety disorder, mental disorder, life quality, sexual dysfunction, sleep disorder, insomnia, anxiety, body image, behavioral symptom, psychological distress, coping behaviors, traumatic stress disorder, breast cancer, and their combination were used. The strategy of searching in English resources was that, in the ADVANCED section of the database, the keyword of Iran in the (AFFILIATION) section and the keyword of breast cancer in the title/abstract section was searched.

### Inclusion and Exclusion Criteria

Inclusion criteria for the study were: Persian and English articles published in domestic and foreign scientific-research journals with full-text available; research should report enough data to calculate the effect size; research should be available in full-text articles online or in an archive of libraries; and articles that report the psychological consequences of breast cancer. Exclusion criteria also included articles not have their full text; studies that refer to breast cancer due to non-psychological factors; studies not reported one of the information needed to calculate the effect size; studies that are not adequate or have serious methodological weaknesses.

### Qualitative Evaluation and Selection of Studies

The content of all articles, after extraction from the desired databases, was evaluated by the PRISMA checklist. Because the analytical unit is the final report of the research conducted on the examined subject in the review studies, in this research, the checklist of the research designs profile designed by Mesrabadi was used to collect the information. The completed checklist was categorized as a coded book (13).

### Statistical Analysis

In this study, meta-analysis method was used to quantitative evaluation of the results. In Cohen’s d index it was chosen as an indicator of the effect size of studies. In order to investigate and analyze the primary research, the effect size of each intervention, the effect size of the combination with two fixed and random effects models, funnel plot, sensitivity analysis, homogeneity test, Chi-I, and NF-S statistics were used. Compressive meta-analysis (CMA) version 2 is used to calculate the effect sizes and the combination of results.

## Results

**Fig. 1:**
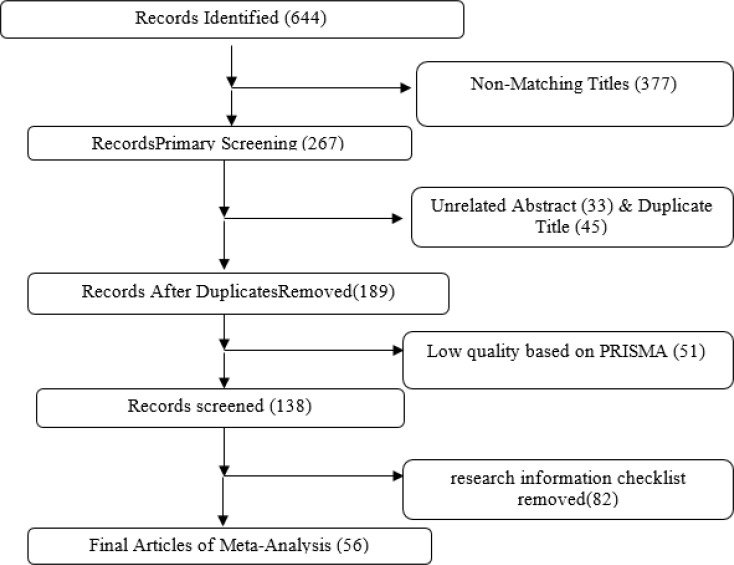
Flow chart of the meta-analysis studies selection

Out of 644 articles obtained, 377 articles that were not consistent with the purpose of the research title were excluded from the study and 267 studies were enrolled for the second phase of screening. The screening criterion in the second stage was unrelated regarding abstract, repetitive articles regarding title and poor quality based on the PRISMA checklist. At that stage, 33 articles were inconsistent with the subject in the abstract, 45 articles regarding repetitive research title and 51 studies were excluded from the study due to lack of minimum qualitative criteria based on the PRISMA checklist. Finally, 138 articles were selected in full text according to the inclusion and exclusion criteria for the final screening and examined based on the research selection checklist, which 82 articles that are lacking the necessary data to enter the meta-analysis were deleted, and finally, 56 articles were included in the meta-analysis study (Fig. 1).

Subsequently, from basic research entered into a meta-analysis, 197 effect sizes were calculated. Two graphics methods (funnel plot) and a statistical index (safe number of destruction) were used to examine the publication bias.

**Fig. 2:**
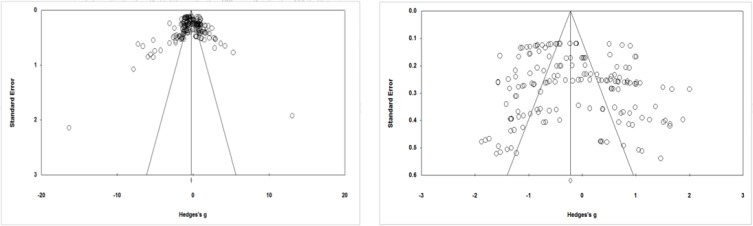
Funnel plot of the publication bias before and after sensitivity analysis

By observing the funnel plot of before sensitivity analysis (Fig. 2), some studies with anomalous and distorted effects have made them asymmetric. Therefore, by removing 32 sizes of the funnel plot after the sensitivity analysis (Fig. 2) is symmetric to the previous graph. Based on the safe number of destruction index, after the entry of 3498 non-significant effect sizes into a meta-analysis, the combined effect sizes calculated will be insignificant. Moreover, the Kendall Tao value was equal to 0.061, and the intercept of the IGER regression was equal to 1.021 which was not significant with the two-tailed test (*P*≤0.05). Thus, by removing 32 extreme effect sizes from 197 initial effect sizes, 165 effect sizes remained, and in subsequent analyses only these effect sizes were used. Moreover, out of 165 residual effect sizes, 122 were significant (*) and 43 were insignificant.

Considering that the main purpose of each meta-analysis is the combination of numerical indices of the primary research in the form of an overall indicator (Table 1), the combined or hedge effect sizes are presented based on two fixed and random models for 165 effect sizes.

**Table 1:** Hedge effect sizes of fixed and random effects related to psychological consequences of breast cancer

***Model***		***Number of effect sizes***	***Hedges effect sizes (g)***	***The standard error***	***Confidence interval 0.95***		***Z Value***	***P***
					***Lower limit***	***Upper limit***		
Before sensitivity analysis	Fixed	197	−0.260	0.016	−0.292	−0.228	−15.836	0.0001
	Random	197	−0.298	0.072	−0.440	−0.156	−4.111	0.0001
After sensitivity analysis	Fixed	165	−0.223	0.017	0.256	−0.190	−13.359	0.0001
	Random	165	−0.132	0.062	−0.253	−0.011	−2.131	0.033

In this meta-analysis, for fixed and random models, the values of the combined effect sizes of 165 effect sizes are respectively −0.223 and −0.132. Both of these effect sizes are statistically significant (*P*≤0.05). To determine the final model of the meta-analysis, the result of the study of the heterogeneity of the effect sizes between the primary researches based on Cochran Q index was 2097.063, which is statistically significant (*P*≤0.01). The results of Chi-I show that over 92% of the variance in the original research results is due to the existence of moderating variables that according to the Higgins, Thompson, Deeks & Altman show the high heterogeneity of the primary researches (14). Based on both heterogeneity indicators, moderating variables play a significant role in the effect sizes of the psychological consequences of breast cancer; therefore, the random model was selected as the meta-analysis model, and the effect size of all studies related to the psychological consequences of the cancer was considered to be −0.132. Moreover, seven areas of psychological consequences of breast cancer were determined as follows according to the results of a meta-analysis (Table 2).

**Table 2:** The effect size of psychological consequences of breast cancer in Iran

***outcome***	***First author***	***Hedges’s g***	***Hedges’s g and 95% CI***				
Anexiety	Overvall Fixed ES	−0.591			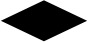		
	Overvall Random ES	−0.762		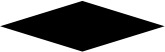			
	Heterogeneity I^2^= 90.855		−2.00	−1.00	0.00	1.00	2.00
Body Image	Overvall Fixed ES	0.072			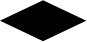		
	Overvall Random ES	0.199			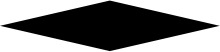		
	Heterogeneity I^2^= 92.730		−2.00	−1.00	0.00	1.00	2.00
Coping strategies	Overvall Fixed ES	0.204			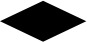		
	Overvall Random ES	0.214			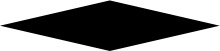		
	Heterogeneity I^2^= 82.219		−2.00	−1.00	0.00	1.00	2.00
Depression	Overvall Fixed ES	−0.567			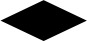		
	Overvall Random ES	−0.700		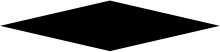			
	Heterogeneity I^2^= 93.249		−2.00	−1.00	0.00	1.00	2.00
Fatique	Overvall Fixed ES	0.187			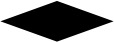		
	Overvall Random ES	0.322			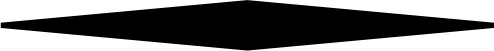		
	Heterogeneity I^2^= 94.541		−2.00	−1.00	0.00	1.00	2.00
Quality Of Life	Overvall Fixed ES	0.073			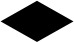		
	Overvall Random ES	0.428			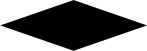		
	Heterogeneity I^2^= 88.428		−2.00	−1.00	0.00	1.00	2.00
Sexual function	Overvall Fixed ES	0.355			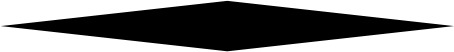		
	Overvall Random ES	0.355			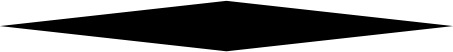		
	Heterogeneity I^2^= 0.000		−2.00	−1.00	0.00	1.00	2.00

## Discussion

This study aimed to identify the psychological consequences of breast cancer. The psychological consequences of breast cancer in women are placed in 7 categories. In fact, the findings of this study can be the summary of the conducted studies that looked at the psychological aspects of breast cancer from the perspective of women with this disease and these categories were the result of a reanalysis of data in a different perspective.

The results of this study indicated that high anxiety and high stress in breast cancer patients were conducted with 36 significant effect sizes and the hedge effect size of 0.762 was considered as the first and most important psychological consequence of breast cancer. Anxiety as one of the most common psychological symptoms in breast cancer patients has the incidence rate of 10% to 30% (15). This state of intense fear, uncertainty, and lack of confidence and excessive fear is considered as an unpleasant response to an unpleasant stimulus. Anxiety can be a cause of fatigue, reduce the efficacy of therapeutic outcomes, and may affect the quality of life and the immune system and neuroendocrine in patients with breast cancer (5). The patient with breast cancer is involved with anxiety symptoms, faced with extreme uncertainty about the future, and concerned about the relapse and side effects of treatment due to predicting negative outcomes caused by the disease.

High depression and low mood in breast cancer patients have been carried out with 30 significant effects sizes and the hedge effect size of 0.700 are considered as the second most psychological consequences of breast cancer in women. The prevalence of depression in cancer patients is 2 to 3 times the general population (16). The main causes of depression in these patients are pain caused by metastasis, reduced social activities, and disability. In patients with breast cancer, common medical treatments also provide a more favorable ground for developing mood disorders, such as depression, because patients experience a condition such as cutting off a member of the body after mastectomy, a member which is a symbol of gender, being a woman and being a mother, and when it is removed, women suffer much stress and may be prone to mood disorders such as depression (17). Since depression is a risk factor in reducing survival rates in cancer patients and is an important factor in patient’s rejection of treatment, timely diagnosis and treatment can be a great help in the treatment of these patients and their rehabilitation and reduce the role of their social activities.

The results of this study indicated that low quality of life in patients with breast cancer with 33 significant effect sizes and the hedge effect size of 0.432 were the third psychological consequence of breast cancer so that these patients report a variety of physical, emotional and social problems. In addition, breast cancer had the greatest impact on the dimensions of social, emotional function and family aspect of patients. About 76% of patients had a moderate decline in their quality of life (18). Moreover, in emotional aspect, the patient faces various psychological problems such as shock, disbelief, and in the social aspect, they face difficulties in interaction and interpersonal relationships (19). Most cancers have toxicities and side effects that severely endanger the quality of life of the patients in the short and long-term (20). Low levels of quality of life in patients are associated with the number of symptoms and complications caused by chemotherapy and the low levels of education of the patient and the family in this regard (21).

Poor sexual function and sexual problems in patients with breast cancer with an effect size of 0.355 were considered as the fourth psychological consequence of breast cancer. In this regard, women treated with mastectomy have a high level of sexual libido decline and more problems with their sexual interests than women with breast-conserving surgery (22). Review of researches on sexuality and breast cancer from 1998 to 2010 has documented a wide range of physical changes in women’s sexuality following breast cancer, including sexual dysfunction such as sexual arousal, lubrication, orgasm and sexual desires (23). In breast cancer, sexual dysfunction can lead to the greatest stress in patients from the time of diagnosis to long-term follow-up, and sexual self-concept can be an important concept in predicting sexual dysfunction.

In the context of the coping strategies used by patients, the use of weak and undesirable emotional adaptive strategies in patients with breast cancer with 13 significant effects sizes and hedge effect size of 0.204 were considered as the fifth psychological consequence of breast cancer. Avoidant coping strategies, a helpless/hopeless coping style with pessimistic or inactive acceptance and submission predict the poor mental compatibility of patients in 1 to 3 years after diagnosis (24). Three main issues of coping with cancer mentioned including emotional disturbances (emotional turmoil and guilty feelings), avoidance (self-distraction and refusal), and rational efforts (cognitive acceptance, threat control & spiritual help-seeking) (25). In addition, training coping skills for these patients is essential to facilitate the transition from emotional coping. The disturbed body image and existence of concerns about body image in patients with breast cancer with 5 significant effect sizes and the hedge effect size of 0.92 were considered as the sixth psychological consequence of breast cancer. The discomfort of body image among women with breast cancer is one of the most common psychological concerns (26). Concerns about the body image are attributed to the lack of breast due to surgery, and the physical changes resulting from received therapies that can affect the various dimensions of women’s lives. Women with the better conceptualization of their body image have better compatibility with cancer, while poor body image usually leads to negative consequences that affect the physical and psychological performance of survivors of breast cancer as well as better relationships with their partner (27). This finding can be justified by using Frank’s demoralization cycle, because in women with breast cancer, due to the conditions with which these patients are confronted, it can lead to disorder in the mental image of the body, stress and continuous mental stress. When mental stress persists for a long time, it provides grounds for adjustment disorders, inability to control emotions, anxiety and depression and other disorders in mental health (28).

Finally, high fatigue in breast cancer patients with 9 significant effect sizes and the hedge effect size of 0.187 were considered as the seventh psychological consequence of breast cancer in women. About 78% of patients expressed fatigue signs and symptoms in varying degrees (29). Cancer-related fatigue has the most discomfort to patients and has the most negative impact on the performance of their lives (30). The underlying mechanism of chronic fatigue in patients with breast cancer is unknown, but both physical and psychological factors are associated with fatigue in patients with breast cancer. Three basic factors involved in fatigue in cancer patients include individual experiences (physical impairment and mental stress), stages of treatment (social constraints and their effects on quality of life) and compatibility with the disease (31).

### Limitations

The failure to differentiate the patients with mastectomy breast cancer, patients undergoing breast conservation surgery (lumpectomy), and patients undergoing radiotherapy in the studies, was the fundamental limitation of the study, which led to not separating the effect sizes of the studies according to the type of patients. Failure to differentiate the stage of the disease in research to further clarify the psychological consequences of breast cancer was another limitation of the study. Since only a few studies have been conducted, on sexual function of women with breast cancer in Iran, further research is recommended. In addition, considering the heterogeneity of studies in the field of quality of life and body image, more research is done on identifying the modalities these two issues in order to provide effective psychological interventions for improving quality of life and body image. Considering the obtained results of the high level of psychological consequences of breast cancer in women with breast cancer, appropriate treatment protocols be designed to adjust the proposed psychological consequences, and its effectiveness in different samples to be examined.

## Conclusion

Breast cancer is the most common cancer among women all over the world. The number of women had breast cancer has increased dramatically in recent years. Breast cancer raises many challenges in women. Exposure to breast cancer itself as a stressful incident can endanger the various aspects of physical, mental and family health. Considering the results of the high level of psychological consequences of breast cancer in women with breast cancer, it is essential to develop appropriate treatment protocols in order to adjust the psychological consequences.

## Ethical considerations

Ethical issues (Including plagiarism, informed consent, misconduct, data fabrication and/or falsification, double publication and/or submission, redundancy, etc.) have been completely observed by the authors.
